# Laparoscopy in bilio-pancreatic surgery in elderly

**DOI:** 10.1186/1471-2318-11-S1-A46

**Published:** 2011-08-24

**Authors:** S Perrotta, V Desiato, O Mazzei, GL Benassai, G Quarto, G Benassai

**Affiliations:** 1 Dipartimento Universitario di Chirurgia Generale, Geriatrica, Oncologica e Tecnologie Avanzate chool of Medicine, University Federico II, Naples, Italy

## Background

More minimally invasive techniques are currently available for the surgical oncologist in the optimal staging of biliopancreatic cancer. With the increased age of population we can see an increase of age-related diseases, such a cardiovascular disease, hypertension, arthritis and other malignancies. With the development of endoscopy, laparoscopy, ultrasonography and biopsy equipment, more minimally invasive techniques are currently available for the surgical oncologist to provide a better optimal diagnosis and strategy, followed by an appropriate surgical treatment of biliopancreatic cancer.

Improvement in the diagnostic and surgical care of elderly cancer patients will have a final impact on disease and overall survival rates of the different types of cancer treatment.

## Materials and methods

We retrospectively reviewed the records of patients between January 2001 and December 2008 who had either a mass in the biliopancreatic area classified as clinically resectable. Tumours were considered to be resectable when there was no evidence of distant extra pancreatic disease or involvement of lymphnodes outside the classic margins of resections. Occlusion or encasement of the superior mesenteric artery or vein, celiac artery or portal vein were used as a criteria for unresectability. Twenty-eight patients over 65 and under 75 years (middle age 69) with primary biliopancreatic cancer were submitted to operations for potentially operative resection. In all cases staging laparoscopy was performed just prior to planned open exploration and resection.

## Results

Twenty seven patients underwent laparoscopy exploration for potential resection. Two of five patients (40%) with distal cholangiocarcinoma survived at 5 years after DCP. Eighteen patients (66.6%) had unresectable disease identified at laparoscopy and fourteen were able to convert to laparotomy palliative surgery while for the others laparoscopy spared an unnecessary laparotomy. In four patients it was possible to perform a laparoscopy palliative surgery.

## Conclusions

Laparoscopy may have a role in the staging of patients with biliopancreatic malignancies, in particular, for reduction of unnecessary exploratory laparotomy.

Moreover, even in old age, duodenocephalopancreasectomy, properly planned and executed, resulted in reduction of operative mortality and morbility and a clear long-term survival.
